# Protocol of the Cologne Corona Surveillance (CoCoS) Study– a prospective population-based cohort study

**DOI:** 10.1186/s12889-021-11206-9

**Published:** 2021-07-02

**Authors:** Max Oberste, Lynn-Marie Pusch, Rebecca Roth, Kija Shah-Hosseini, Felix Dewald, Claudia Müller, Luise Stach von Goltzheim, Clara Lehmann, Michael Buess, Anna Wolff, Gerd Fätkenheuer, Gerhard Wiesmüller, Florian Klein, Martin Hellmich, Florian Neuhann

**Affiliations:** 1 Institute of Medical Statistics and Computational Biology, Medical Faculty and University Hospital of Cologne, University of Cologne, Robert-Koch-Straße 10, 50931 Cologne, Germany; 2 Institute of Virology, Medical Faculty and University Hospital of Cologne, University of Cologne, Fürst-Pückler-Straße 56, 50935 Cologne, Germany; 3 Department of Internal Medicine, Medical Faculty and University Hospital of Cologne, University of Cologne, Kerpener Str. 62, 50931 Cologne, Germany; 4 Cologne Health Authority, Cologne, Germany; 5 Heidelberg Institute of Global Health, University Heidelberg, Heidelberg, Germany; 6 School of Medicine and Clinical Sciences, Levy Mwanawasa Medical University, Lusaka, Zambia

**Keywords:** Coronavirus, COVID-19, Pandemic, Surveillance, Socio-economic factors, Risk behaviours, Rolling cohort

## Abstract

**Background:**

Surveillance strategies are critical to cope with the current SARS-CoV-2 pandemic and to evaluate, as well as adjust government-imposed countermeasures. Incidence estimates are widely based on laboratory confirmed cases reported by health authorities. Prevalence and incidence data of SARS-CoV-2 is still scarce, along with demographic and behavioural factors associated with infection risk.

**Methods:**

The Cologne Corona Surveillance Study will be conducted in the City of Cologne, which is the fourth-largest city in Germany with a population of approximately 1.1 million. Researchers will apply self-sampling surveillance to a rolling cohort of Cologne residents. Random samples of 6000 Cologne residents 18 years of age and older will be drawn from the registration office. Upon receiving the information and saliva sample kit, participants will be asked to fill out a questionnaire online or via phone, sign written informed consent, and send back written consent, as well as saliva sample. The saliva samples will be tested for SARS-CoV-2 by reverse PCR. The questionnaire will be administered to gather information about personal characteristics such as health status and risks. A second round of testing will take place 6 weeks after the first.

**Discussion:**

Self-administered saliva sampling proved to be a legitimate and feasible alternative to nasopharyngeal swabs taken by health professionals. However, it is unclear whether the targeted response rate of 40% can be achieved and whether the results are representative of the population.

**Trial registration:**

DRKS.de, *German Clinical Trials Register* (DRKS), Identifier: DRKS00024046, Registered on 25 February 2021.

## Background

The first detection of Severe Acute Respiratory Syndrome-Coronavirus-2 (SARS-CoV-2) in December 2019 in Wuhan, China, and its subsequent worldwide spread in spring 2020 signalled the beginning of a global pandemic and public health crisis [1]. As of April 13th 2021, the virus had infected over 135 million people, resulting in 2.9 million deaths [2]. Germany’s public health authorities report more than 3 million cases and almost 79,000 deaths on April 13th. The 7-day notification rate of newly reported SARS-CoV-2 infections has risen to more than 160 cases per 100,000 inhabitants [3], which poses a serious threat to hospitals and intensive care units. Facing the pandemic, the German federal government imposed countermeasures including lockdown for economy and private households, the quarantine of confirmed patients and their direct contacts, the mandatory use of personal protective equipment, the implementation of social distancing with restrictions of private gatherings and closure of public and private institutions including schools and kindergartens [4]. The countermeasures result in a significant reduction in incidence, but at the cost of serious psychological [5], social [6], and economic consequences [7].

The current incidence estimates in Germany are based on laboratory confirmed cases reported by health authorities, which are influenced by availability of tests and acceptance of these tests. As a result, it is likely that underreporting will occur because a noticeable number of SARS-CoV-2 infections are asymptomatic or mild. This is important because asymptomatic and mild cases are infectious and play an important role in transmission [8]. To better understand the spread of the virus and to provide a valid data base for effective countermeasures, effective surveillance strategies beyond laboratory confirmed cases are needed. Population-based studies have already been launched in Berlin [9] and Munich [10], as well as in Bonn [11].

This protocol introduces the ‘Cologne Corona Surveillance Study’. The prospective, representative, and rolling cohort will receive saliva sampling kits via mail and their questionnaires on socioeconomics and health issues will be completed online or over the phone two times, 6 weeks apart. Saliva samples will be sent to our testing facility for analysis of SARS-CoV-2.

## Methods

### Aim

The aims of CoCoS are:

1. To provide a more precise estimate of the prevalence and incidence of SARS-CoV-2 in the general population of Cologne by estimating the extent of undetected SARS-CoV-2.
2. To estimate differences in prevalence and incidence by age, gender, housing characteristics, and socioeconomic status.
3. To study the relationship between behavioural factors (risk behaviour, working from home or going to work, using public transportation) and risk perception with SARS-CoV-2 infections.
4. To compare the results with other regional population-based studies [9–11] in order to discover optimal surveillance strategies for COVID-19 outbreaks, as well as geographic predictors.

### Setting

The City of Cologne is situated in the West of Germany, near the borders between Germany and the Netherlands, Belgium and Luxembourg. With a population of approximately 1.1 million, Cologne is the fourth-largest city in Germany and the largest city of the federal state of North Rhine-Westphalia. Around 18% of the population in Cologne count among the particularly vulnerable age groups for a severe COVID-19, 12.1% are 65 to 80 years and another 5.4% are older than 80 years [12]. The population density is 2697 people per square kilometre [13]. There are 68 hospital beds and 27 doctors per 10,000 people [14].

The SARS-CoV-2 outbreak spread from the district of Heinsberg, 64 km west of Cologne, during carnival celebrations and towards the end of winter holiday season. As the local countermeasures did not work, a state-wide countermeasure was implemented by the state government of North Rhine-Westphalia [15–18]. A nationwide lockdown was imposed March 22nd, 2020 [4] in order to control the spread of the virus. Locally, in the City of Cologne, additional measures comprised mask regulations, prohibitions on consumption of alcohol between 3 p.m. and 6 a.m. and restrictions on street art posted in specific public places [19]. Throughout spring and summer 2020, step-by-step nationwide measures were lifted as a result of lower incidence. As the incidence numbers rose again (second wave) in 2020, a second lockdown was imposed 2nd of November 2020. Since the numbers would continue to rise, these measures would be expanded on December 16th, 2021. A nationwide vaccination campaign was launched on December 26th, 2020. However, despite measures to control the spread of the virus and despite the beginning vaccination campaign, there were 38,565 cases registered in Cologne until mid-April 2020, and 610 died from or with SARS-CoV-2 [20]. By March and April 2021, incidence reached 160 confirmed cases within 7 days and remained high [3] (third wave). The German health system is at risk of becoming overburdened with the growing number of severe SARS-CoV-2 cases. At the end of April 2021, of the 448 intensive care units in Cologne, only 29 beds were available for patients who suffered from SARS-CoV-2; out of these, 65 needed mechanical ventilation [21].

### Study design

**Fig. 1:**
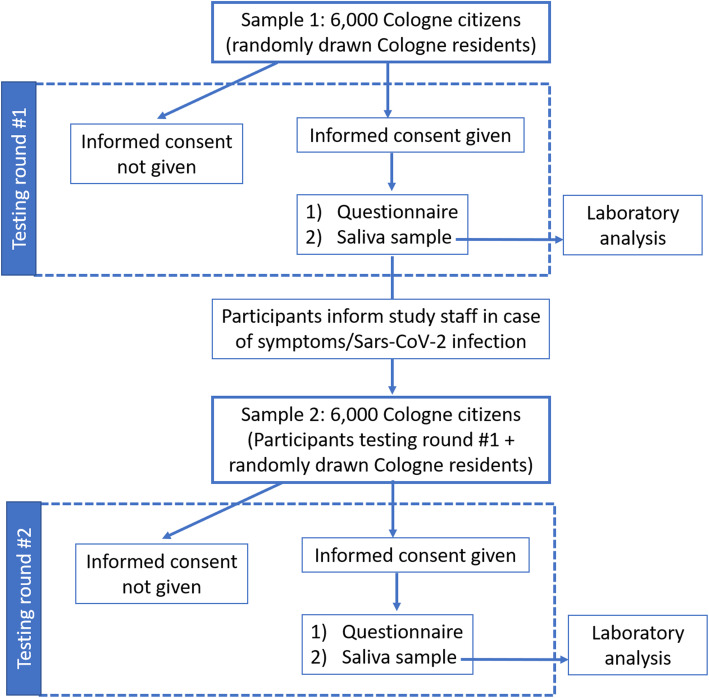
Flow chart of enrolment and testing at each surveillance round

The study will be conducted as a prospective, longitudinal, surveillance cohort study, involving at least two rounds of testing with a representative sample of citizens of the City of Cologne (*n* = 6000). The study flowchart is shown in Fig. 1. The study protocol was approved by the Ethics Committee of the Medical Faculty of the University of Cologne and by the Ethics Committee of the North Rhine Medical Association. Study details were added to the German Clinical Trial Register (GermanCTR) (Identifier: DRKS00024046). Any modifications to the protocol, which may impact on the conduct of the study, potential benefit of the patient or may affect patient safety, including changes of study objectives, study design, study population, sample sizes, study procedures, or significant administrative aspects will require a formal amendment to be approved by both Ethics Committees.

### Sample

Inclusion criteria comprise of residence in the City of Cologne and age of 18 years or older. Exclusion criteria comprise of age younger than 18, living in a closed institution, being imprisoned, or unable to give consent. Initially, a random sample of 6000 Cologne citizens will be drawn from the registration office using a random generator in the official registration management program (MESO, HSH Soft- und Hardware Vertriebs GmbH, 16,356 Ahrensfelde OT Lindenberg). Participants will be assigned a unique study ID and will receive detailed information about the study, including the study’s objectives, details about participation, and information about data storage, as well as information about confidentiality. Participation is voluntary, and selected citizens will be free to decline participation at any time with no adverse consequences. Participants in the study will be only included with written informed consent. Participants who will decline informed consent in the first testing round will be replaced in order to reach again 6000 potential participants. The newly invited participants will be drawn from a second sampling from the registration office as described above.

### Study procedures

Figure 1 shows the surveillance strategy that is planned to be implemented. The 6000 randomly selected Cologne citizens will be notified by regular mail, with all envelopes being sent out simultaneously. Besides the invitation letter, a saliva sample and a pre-addressed, stamped UN3373 envelope will be included in the package. Participants will be required to give written informed consent and to send a saliva sample within 1 week of receiving the package to the Institute of Virology, University Hospital Cologne. On the informed consent form, participants will record the exact date of the saliva sampling.

At the University Hospital of Cologne, the existence of a written informed consent will be checked, saliva samples will be analysed and participants who test positive will be notified immediately. Participants will also be provided with a QR-code and a link for completing the study questionnaire online, as well as a phone number to call study staff in case they need assistance. Participants can choose to complete the questionnaire during a telephone interview with a member of the study team. All participants are asked to inform study staff if they experience symptoms between testing rounds. A second testing round will take place 6 weeks after the first. Data on non-participation will be collected along with demographic and socioeconomic information of non-responders, as well as reasons for non-participation. In order to assess representativeness, the data will be compared with official statistics of the City of Cologne.

### Study instruments

#### Questionnaires

The first round of the CoCoS study survey will be conducted using a standard questionnaire that includes questions about personal data, such as age and health status, risk behaviours, and potential sources of infection. Therefore, we can detect possible sources of infection, as well as assess socioeconomic factors in our participants. We also hope to explore potential correlations between infection status and socioeconomic factors.

The CoCoS questionnaire is aligned with the ELISA and MuSPAD questionnaires developed by the ‘Helmholtz Zentrum für Infektionsforschung’ [22]. The questionnaire was then adjusted for the specific focus of the CoCoS study, consisting of five starting questions, plus 23 main questions in the first round and 17 questions in the second round, to which follow-up questions were added based on the selected answers. Several sections are included in the questionnaire, including personal information, housing conditions, household members, education and employment, possible symptoms, past SARS-CoV-2 test results, preventive behaviours and possible sources of infection. The CoCoS questionnaire is estimated to take from 5 to 10 min to complete.

Participants will be asked to fill out their specific ID at the beginning of each interview in order to ensure that only our study members can access the questionnaire and to be able to match their sample to the interview.

The second round of the questionnaire will examine issues that may have changed from round one to round two.

#### Laboratory analysis

Samples will be analysed and stored at the University of Cologne, Faculty of Medicine and University Hospital Cologne, Institute of Virology. The samples will be stored at 4 °C upon arrival until further processing. The salivettes will be centrifuged at 1000 x g for 2 min. One-hundred μl of saliva will be used for subsequent RT-qPCR in a pool of each 10 samples, containing 1 ml of pooled saliva. For SARS-CoV-2 detection, either the COBAS 6800 (Roche Diagnostics) or Alinity (Abbott) instruments equipped with their respective SARS-CoV-2 detection kits will be used. One ml of each sample of a positive pool will be re-tested individually as described above.

For analysis of the variants of SARS-CoV-2 positive samples, 500 μl of saliva will be used to purify nucleic acids with the MagNa Pure 96 automatic nucleic acid extraction instrument and the Viral NA large volume Kit. One-hundred μl will be used for elution. Of the extracted RNA, 5 μl will be used in a qPCR using the VirSNip SARS-CoV-2 Spike A23063T N501Y, del21765–770 del HV69/70 and G23012A E484K assay according to the manufacturer’s instructions (TIBMolBiol, Berlin, Germany). Melting analyses will be performed in a LightCycler® 480 II (Roche Diagnostics).

### Statistics

#### Sample size calculation

With a true prevalence of [1; 2; 5; 1%; 2%; 5%] and an expected sample size of *n* = 2500 participants, a precision of the estimate of [±1; ±2; ±3; ±4; ±5; ±9] (i.e., the width of a 95% confidence interval) is to be achieved, which is considered sufficient. Note, on April 9th, 2021, SARS-CoV-2 prevalence of confirmed cases in Cologne was 3.9% [20].

#### Analysis

The distributions of the collected data will be first described with the usual parameters of location and distribution, i.e. mean, standard deviation, percentiles (0., 25., 50., 75., 100.) for continuous variables, absolute and relative frequencies for qualitative variables.

Associations and correlations will be described by means of contingency tables and regression methods (e.g. logistic regression for dichotomous target variables). With regard to the prevalence of SARS-CoV-2, the following variables are of particular interest: age, gender, neighbourhoods, housing size, number of household members, pre-existing conditions, previous positive testing, potentially contagious contacts. Where possible, important statistical measures will be provided with 95% confidence intervals to indicate the precision of the estimate.

Due to the expected limited participation rate (we estimate 40%) studies of the representativeness of the sample obtained are of particular importance. On the basis of the information provided by the non-participants, an extrapolation to the Cologne population will be carried out. (Notabene: The risk of non-participation due to lack of reading and language skills should be minimized by the assistance of native speakers. Otherwise, a bias correction will be attempted as described below.) Official statistics on the total population of Cologne are available at https://www.stadt-koeln.de/politik-und-verwaltung/statistik/ (accessed 22.01.2021). Sensitivity analyses examine the correction for various bias constellations. For this purpose, corresponding weights will be used in combination with regression methods.

The calculations will be performed using scripts for the programming languages R (R Foundation for Statistical Computing, Vienna, Austria), SAS (SAS Institute Corp., Cary, NC, USA), Stata (StataCorp LLC, College Station, TX, USA) and SPSS Statistics (IBM Corp., Armonk, NY, USA).

### Data management

An instance of the Internet application REDCap (Research Electronic Data Capture) (Vanderbilt University, Nashville, TN, USA) hosted at the Clinical Trials Center Cologne will be used to record the questionnaires. A single MySQL database will store all of REDCap’s data and associated system and project information. This open source relational database management system makes use of foreign keys and indexes to accommodate the project and system data.

## Discussion

Despite the importance of surveillance for the current SARS-CoV-2 pandemic, specific data on prevalence and incidence of SARS-CoV-2, the association between risk of infection and demographic factors, as well as behavioural factors, are still scarce in Germany. This information can be used to identify risk factors for infections, better understand how the virus spreads and evaluate the efficacy and efficiency of public health measures. A cohort study can provide these data. The rolling cohort in Cologne will provide a representative sample.

The CoCoS study presents a self-sampling strategy as a surveillance strategy. This approach allows us to reach a large and representative sample of Cologne citizens and it is less labour-intensive than taking samples from mobile testing sites [23] or general practitioners [24]. It is crucial that response rates are high using self-sampling, as low participation rates negatively affect the generalizability of results. With support from the Cologne authorities and a media announcement, we are hopeful that 40% of the 6000 residents invited for participation will respond.

Our self-sampling method of choice was saliva swab, which is particularly easy to handle for participants, and is comparable to the gold standard in the diagnosis of acute infections with SARS-CoV-2, nasopharyngeal swab [25].

The Cologne Corona Surveillance Study will consider several factors to ensure that responses do not correlate with acute infections. A self-sampling design will allow residents who have acute symptoms or who are under quarantine to participate in the study. In order to ensure representativeness of the study results, self-sampling and questionnaires can be completed at home and are time-free. This allows residents who are unable to visit a test centre during regular working hours (e.g. working hours) to participate.

One-week response time should ensure comparability of epidemiological background. A simple study description with step-by-step instructions was used, as well as an accessible phone number for assistance with self-sampling kits and questionnaires to reach residents with as wide a range of experiences as possible.

Nevertheless, our study is not without limitations. We cannot rule out the possibility that people with little computer experience or no Internet access might be less likely to complete the questionnaire. This is particularly relevant in the case of older residents. It is possible that older participants may not be able to provide consent to participate in our study or may struggle with the administrative challenges to participate. Therefore, the representativeness of data from older participants in our study must be critically analysed. In addition, foreign-born residents who do not speak German as their first language may have difficulties understanding the invitation letter and refrain from participating in the study. Further, our study does not include residents under 18 years of age and will not detect SARS-CoV-2 positive individuals currently in intensive care.

## Data Availability

Not applicable.

## Abbreviations

CoCoS-study: Cologne Corona Surveillance Study
SARS-CoV-2: Severe Acute Respiratory Syndrome-Coronavirus-2
PCR: Polymerase Chain Reaction
DRKS: German Clinical Trials Register
COVID-19: Corona Virus Disease 19
RT-qPCR: Quantitative reverse transcription PCR
RNA: Ribonucleic Acid
